# Ribosomal Protein S6 Phosphorylation in the Nervous System: From Regulation to Function

**DOI:** 10.3389/fnmol.2015.00075

**Published:** 2015-12-16

**Authors:** Anne Biever, Emmanuel Valjent, Emma Puighermanal

**Affiliations:** ^1^Centre National de la Recherche Scientifique, UMR5203, Institut de Génomique FonctionnelleMontpellier, France; ^2^Institut National de la Santé et de la Recherche Médicale, U1191Montpellier, France; ^3^Université de Montpellier, UMR-5203Montpellier, France

**Keywords:** rpS6 phosphorylation, mRNA translation, ribosome, mTOR, S6K, PP-1, brain, signaling cascades

## Abstract

Since the discovery of the phosphorylation of the 40S ribosomal protein S6 (rpS6) about four decades ago, much effort has been made to uncover the molecular mechanisms underlying the regulation of this post-translational modification. In the field of neuroscience, rpS6 phosphorylation is commonly used as a readout of the mammalian target of rapamycin complex 1 signaling activation or as a marker for neuronal activity. Nevertheless, its biological role in neurons still remains puzzling. Here we review the pharmacological and physiological stimuli regulating this modification in the nervous system as well as the pathways that transduce these signals into rpS6 phosphorylation. Altered rpS6 phosphorylation observed in various genetic and pathophysiological mouse models is also discussed. Finally, we examine the current state of knowledge on the physiological role of this post-translational modification and highlight the questions that remain to be addressed.

## Introduction

The eukaryotic ribosome is composed of the small 40S and the large 60S subunits, comprising together 4 ribosomal RNA species and 79 ribosomal proteins (Kressler et al., 2010). In many organisms, ribosomal proteins undergo various post-translational modifications, including phosphorylation, acetylation, methylation, *O*-linked β-*N*-acetylglucosaminylation, and ubiquitylation (Xue and Barna, 2012). Historically, the phosphorylation of the 40S ribosomal protein S6 (rpS6) was the first post-translational modification described (Gressner and Wool, 1974). The presence of phospho-rpS6 (p-rpS6) at different levels in a 2D gel provided the first evidence that rpS6 phosphorylation could occur at several residues (Lastick et al., 1977). Ensuing studies identified five evolutionary conserved and clustered carboxy-terminal phospho-sites, which undergo phosphorylation in an ordered manner, beginning with Ser236 and followed sequentially by Ser235, Ser240, Ser244, and Ser247 (Martin-Pérez and Thomas, 1983; Wettenhall et al., 1992; Meyuhas, 2008, 2015). Intriguingly, the exact function of the post-translational modification of this indispensable ribosomal protein remains enigmatic. Despite the large debate regarding its physiological role, rpS6 phosphorylation is commonly used as a marker for neuronal activity and a readout of mammalian target of rapamycin complex 1 (mTORC1) activity (Meyuhas, 2008, 2015; Mahoney et al., 2009; Knight et al., 2012). This review summarizes our current knowledge regarding the molecular mechanisms as well as the variety of stimuli modulating rpS6 phosphorylation in the nervous system.

## Regulation of rpS6 phosphorylation

**Figure 1:**
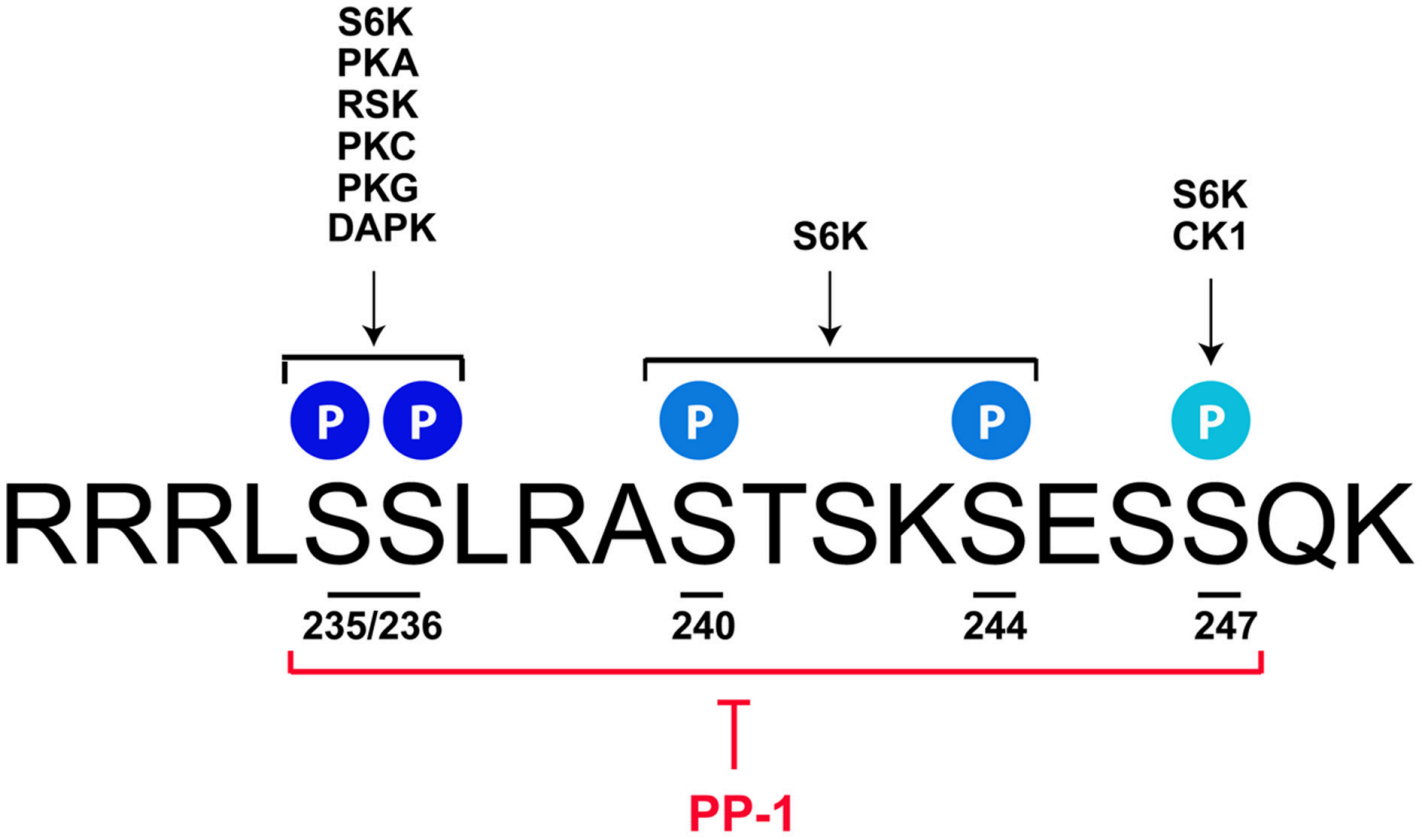
**rpS6 phosphorylatable residues are targeted by multiple kinases and dephosphorylated by PP-1**. *Mus musculus* sequence of the C-terminal domain of rpS6 depicting the 5 phosphorylatable sites and their respective kinases. S6K catalyzes the phosphorylation of all the residues, while PKA, RSK, PKC, PKG, and DAPK target the S235 and S236 sites. CK1 selectively phosphorylates the S247 residue. All phospho-sites are dephosphorylated by PP-1. *See text for details*.

The p70/p85 S6 kinase 1 (S6K1), which is able to catalyze the phosphorylation of rpS6 at all sites, was the first kinase identified (Krieg et al., 1988; Ferrari et al., 1991; Bandi et al., 1993; Meyuhas, 2008, 2015). Further studies described additional protein kinases targeting selectively the Ser235 and Ser236 residues. These include p90 Ribosomal S6 Kinases (RSK1-4) (Roux et al., 2007), Protein Kinase C (House et al., 1987), Protein Kinase A (PKA) (Moore et al., 2009; Valjent et al., 2011; Yano et al., 2014; Biever et al., 2015), Protein Kinase G (Yano et al., 2014), and Death-Associated Protein Kinase (DAPK) (Schumacher et al., 2006) (Figure 1). Although less studied, the residue Ser247 has been identified as a target of Casein Kinase 1 (Hutchinson et al., 2011). Contrasting with the diversity of kinases regulating rpS6 phosphorylation, the dephosphorylation of the five residues is achieved by a single phosphatase: the Protein Phosphatase-1 (PP-1) (Belandia et al., 1994; Hutchinson et al., 2011) (Figure 1). Since the molecular mechanisms regulating rpS6 phosphorylation have been recently extensively reviewed (Meyuhas, 2015), we focus on the contribution of S6K1/2 kinases and the PKA/PP-1 pathway, being the main upstream mechanisms described to regulate rpS6 phosphorylation in the nervous system.

### S6K1/2 pathway

In mammalian cells, two different genes encode two isoforms of the S6 Kinase, S6K1, and S6K2. S6K1 has cytosolic and nuclear isoforms (p70 S6K1 and p85 S6K1, respectively), whereas both S6K2 isoforms (p54 S6K2 and p56 S6K2) are primarily nuclear (Martin et al., 2001). As demonstrated by the use of S6K1/S6K2 double knockout mice, both isoforms contribute to the regulation of basal and inducible rpS6 phosphorylation at S235/236 and S240/244 sites (Pende et al., 2004; Kroczynska et al., 2009; Chauvin et al., 2014). Different observations were made in single S6K knockout mice. Indeed, while the S6K2 knockout mice display a reduction of rpS6 phosphorylation only at S235/236 sites in the hippocampus (Antion et al., 2008a), S6K1-deficient mice show no alterations (Antion et al., 2008a; Bhattacharya et al., 2012). Compensatory mechanisms taking place in the single knockout mice could explain these latter observations since viral-mediated overexpression of a constitutive-active S6K1 (S6K1 CA) or kinase-inactive S6K1 (S6K1 KI) in the medial prefrontal cortex increases or decreases basal rpS6 phosphorylation, respectively (Dwyer et al., 2015). Pharmacological evidences also support the critical role of S6K in the regulation of rpS6 phosphorylation. S6K1/2 undergo phosphorylation at 8 Ser/Thr phospho-sites, including 4 serine residues in the C-terminal autoinhibitory domain. Phosphorylation of the autoinhibitory domain was originally proposed to trigger a more relaxed conformation of the protein allowing its phosphorylation at T389 by mTORC1 leading to S6K activation (Dennis et al., 1998). Thus, the blockade of canonical mTORC1/S6K signaling by the mTORC1 inhibitor rapamycin suppresses both basal and stimuli-induced rpS6 phosphorylation in various brain areas (Kelleher et al., 2004; Takei et al., 2004; Cota et al., 2006; Antion et al., 2008a; Gobert et al., 2008; Géranton et al., 2009; Santini et al., 2009; Zeng et al., 2009; Huang et al., 2010; Cao et al., 2011; Troca-Marín et al., 2011; Wu et al., 2011; Bailey et al., 2012; Bertran-Gonzalez et al., 2012; Meffre et al., 2012; Brewster et al., 2013; Macias et al., 2013; Bowling et al., 2014; Biever et al., 2015).

Although the activation of S6K and the subsequent phosphorylation of rpS6 are commonly used as a readout of mTORC1 activation, several evidences point out the existence of a synergistic crosstalk between mTORC1 and the extracellular signal-regulated kinase (ERK) signaling to control rpS6 phosphorylation. Thus, ERK can promote S6K activation by enhancing its phosphorylation at T421/S424 sites (Mukhopadhyay et al., 1992). When phosphorylated, these sites located in the autoinhibitory domain of the S6K1/2 are thought to prime the activation of S6K, thereby facilitating the subsequent phosphorylation of the other sites of S6K by the upstream kinases (Dennis et al., 1998). Alternatively, ERK can also modulate the mTORC1/S6K cascade upstream of S6K. On the one hand, ERK-mediated inhibitory phosphorylation of Tuberous Sclerosis Complex 2 (TSC2) stimulates the Ras Homolog Enriched in Brain (Rheb) protein, which in turn activates mTORC1 (Roux and Blenis, 2004; Long et al., 2005; Ma et al., 2005). On the other hand, ERK can enhance mTORC1 activation through RSK-mediated phosphorylation of Raptor (Wettenhall et al., 1992). Examples of this synergistic interaction between ERK and mTORC1 in the regulation of rpS6 phosphorylation have been reported in several models and in various brain areas (Kelleher et al., 2004; Antion et al., 2008a; Gobert et al., 2008; Santini et al., 2009; Gangarossa et al., 2014) Interestingly, ERK can also regulate rpS6 phosphorylation at S235/236 through RSK independently of mTORC1/S6K signaling (Roux et al., 2007). Indeed, the increase in pS235/236-rpS6 promoted by tetraethylammonium in cultured cortical neurons is prevented by RSK3 inhibition (Gu et al., 2015). Finally, a recent study performed in hippocampal neurons demonstrated that the cdk5-dependent phosphorylation of S6K at S411 site is also critical in the regulation of S6K activation and the subsequent rpS6 phosphorylation at S235/236 sites (Lai et al., 2015).

### cAMP/PKA pathway

The enhanced rpS6 phosphorylation in the cerebral cortex following the administration of *N6O-2*′dibutyryl cAMP was one of the first demonstrations that cAMP could modulate *in vivo* the state of phosphorylation of rpS6 (Roberts and Morelos, 1979). Despite this evidence, the contribution of the cAMP/PKA pathway in the regulation of rpS6 phosphorylation in the nervous system has been largely neglected. However, recent studies highlighted the importance of cAMP/PKA signaling in the regulation of rpS6 phosphorylation at S235/236 sites. Thus, the direct stimulation of the adenylate cyclase by forskolin increases pS235/236-rpS6 in the striatum and the hippocampus (Gobert et al., 2008; Biever et al., 2015) (Table 1). Similar results are obtained when the degradation of cAMP is prevented by the administration of papaverine, a potent inhibitor of type 10A phosphodiesterase (Biever et al., 2015). Although the demonstration that PKA directly catalyzes rpS6 phosphorylation in the brain is still lacking, several indirect evidences support its contribution in the control of pS235/236-rpS6 following cAMP elevation. Indeed, forskolin-induced rpS6 phosphorylation in striatal slices is reduced in the presence of a PKA inhibitor (Biever et al., 2015). Moreover, stimulation of PKA activity with the cAMP analog cBIMPS increases pS235/236-rpS6 in striatal culture (Valjent et al., 2011) (Table 1). Finally, the administration of pharmacological agents promoting PKA activation triggers rpS6 phosphorylation in several brain areas (Gobert et al., 2008; Valjent et al., 2011; Knight et al., 2012; Bonito-Oliva et al., 2013; Rapanelli et al., 2014; Biever et al., 2015; Sutton and Caron, 2015).

**Table 1:** **Pharmacological stimuli modulating rpS6 phosphorylation *ex vivo***.

**Brain areas**	**Model**	**Treatment**	**S235/236**	**S240/244**	**References**
**WHOLE BRAIN**					
	Culture	Insulin	↑ (NS)	↑ (NS)	Heidenreich and Toledo, 1989
**HIPPOCAMPUS**					
	Culture	BDNF	↑	ND	Kelleher et al., 2004; Troca-Marín et al., 2011
		Bicuculline	↑	ND	Kelleher et al., 2004
	Slice	DHPG	↑	↑	Antion et al., 2008a
		Forskolin	↑	ND	Gobert et al., 2008
**STRIATUM**					
	Culture	cBIMPS	↑	ND	Valjent et al., 2011
		Haloperidol	ND	↑	Bowling et al., 2014
	Slice	Forskolin	↑	ND	Biever et al., 2015
		6-OHDA/SKF81297	↑	↑	Santini et al., 2009
**CORTEX**					
	Synaptoneurosomes	BDNF	ND	↑	Takei et al., 2004
	Culture	Leucine	ND	↑	Ishizuka et al., 2008
		Bicuculline/glycine	↑	ND	Lai et al., 2012
		Tetraethylammonium	↑	ND	Gu et al., 2015
		BDNF	ND	↑	Lenz and Avruch, 2005
		GNA	ND	↑	Lenz and Avruch, 2005
		Glutamate/NMDA	ND	↓	Lenz and Avruch, 2005
		Bicuculline/4-AP	ND	↑	Lenz and Avruch, 2005

*NS, not specified; ND, not determined; GNA, Glutamate + NMDA followed 5 s later by the NMDA antagonist APV; 4-AP, 4-aminopyridine*.

Although PKA targets selectively S235/236 residues (Moore et al., 2009), recent evidences suggest that PKA also contributes indirectly to rpS6 phosphorylation through a protein phosphatase cascade. This mechanism has been particularly well-studied in the striatum, where the inhibition of PP-1, controlled by the PKA-dependent phosphorylation of dopamine- and cAMP-regulated phosphoprotein, M*r* 32,000 (DARPP-32) at T34 (Hemmings et al., 1984; Greengard, 2001), promotes pS235/236-rpS6 induced by d-amphetamine or haloperidol (Valjent et al., 2011; Bonito-Oliva et al., 2013; Biever et al., 2015). This mechanism also contributes to the regulation of rpS6 phosphorylation at S240/244 sites (Bonito-Oliva et al., 2013). These findings highlight the importance of PKA/DARPP-32/PP-1 signaling in the regulation of rpS6 phosphorylation in the striatum and raise the intriguing hypothesis that similar mechanisms could take place in other brain areas.

## Stimuli modulating rpS6 phosphorylation in the brain

Recently an increasing number of studies have used rpS6 phosphorylation as a marker for neuronal activation in the context of synaptic plasticity or in response to variety of therapeutic agents in physiological and pathophysiological contexts.

### Synaptic plasticity

Increased rpS6 phosphorylation during synaptic plasticity was reported for the first time by Klann and colleagues in 1991 using a synthetic peptide of rpS6 containing the residues 222–249. Since then, enhanced rpS6 phosphorylation has been observed in several electrical or chemical models of synaptic plasticity. Thus, pS235/236-rpS6 increases in the CA1 subfield of the hippocampus during long-term potentiation following high frequency stimulation (Antion et al., 2008b) or forskolin application (Kelleher et al., 2004; Antion et al., 2008b; Gobert et al., 2008). Similarly, mGluR-dependent long-term depression induced by application of the mGluR1 agonist [3,5-RS] dihydroxyphenylhydrazine (DHPG) is associated with marked increases in pS235/236- and pS240/244-rpS6 in hippocampal slices (Antion et al., 2008a). Interestingly, a recent study reported that the state of phosphorylation of rpS6 at S240/244 sites could be used to estimate the neuronal activity state of striatal cholinergic interneurons (Bertran-Gonzalez et al., 2012).

### Pharmacological stimuli

A large number of pharmacological stimuli have been described to promote rpS6 phosphorylation in neurons (Tables 1, 2). Indeed, several *ex vivo* studies performed in slices or neuronal cultures showed that rpS6 phosphorylation is enhanced by stimuli triggering multiple forms of neuronal activity (Table 1). *In vivo*, the phosphorylation of rpS6 has been assessed following a single or repeated administration of a large variety of pharmacological agents in various brain areas (Table 2). Thus, proconvulsant drugs such as kainate, pilocarpine, pentylenetetrazol (PTZ), or the dopamine D1R agonist SKF81297 lead to a massive increase in pS235/236- and pS240/244-rpS6 in principal cells in the hippocampus and in various cortical areas (Table 2). Moreover, the administration of drugs of abuse (cocaine, d-amphetamine, methamphetamine, morphine, and tetrahydrocannabinol) as well as antipsychotics (haloperidol, clozapine, and olanzapine) also regulates rpS6 phosphorylation at multiple sites in several brain areas including the striatum, the nucleus accumbens, the cortex, and the hippocampus (Table 2). Finally, several hormones involved in the regulation of energy balance enhance rpS6 phosphorylation in hypothalamic nuclei (Table 2).

**Table 2:** **Pharmacological stimuli modulating rpS6 phosphorylation *in vivo***.

**Brain areas**	**Model**	**Treatment**	**Doses (mg/kg)**	**S235/236**	**S240/244**	**Cell-Type**	**References**
**HIPPOCAMPUS**							
	Mouse	SKF81297	5	↑	=	Granule cells DG	Gangarossa et al., 2011; Gangarossa and Valjent, 2012
			5^*^	↑	↑	Granule cells DG	Gangarossa et al., 2014
		Kainate	12.5	ND	↑	Principal cells	Knight et al., 2012
		THC	10	↑	ND	Pyramidal cells	Puighermanal et al., 2009
	Rat	Kainate	10	↑	ND	Pyramidal cells	Macias et al., 2013
			12	↑ (NS)	↑ (NS)	ND	Zeng et al., 2009
				ND	↑	ND	Chen et al., 2012
		Pilocarpine	300	↑ (NS)	↑ (NS)	ND	Huang et al., 2010
		PTZ	75	↑	↑	ND	Zhang and Wong, 2012
**STRIATUM**							
	Mouse	Cocaine	30	ND	↑	MSNs	Knight et al., 2012
		D-amphetamine	5	↑	=	ND	Rapanelli et al., 2014
			10	↑	=	D1-MSNs	Biever et al., 2015
			10^*^	↑	=	ND	Biever et al., 2015
		Haloperidol	0.5	↑	↑	D2-MSNs	Valjent et al., 2011; Bonito-Oliva et al., 2013
		Clozapine	5	↑	↑	D2-MSNs	Valjent et al., 2011
		Papaverine	30	↑	↑	D1- and D2-MSNs	Biever et al., 2015
		SKF81297	5	↑	ND	D1-MSNs	Gangarossa et al., 2013
		Quinpirole	1	↓	=	D1-MSNs	Gangarossa et al., 2013
		Apomorphine	3	↑	ND	D1-MSNs	Gangarossa et al., 2013
		6-OHDA/L-DOPA	20	↑	↑	D1-MSNs	Santini et al., 2009
			20^*^	↑	↑	D1-MSNs	Santini et al., 2009
			10	↑	↑	D1-MSNs	Santini et al., 2012
	Rat	Quinelorane	0.16	=	↑	ND	Salles et al., 2013
	Monkey	MPTP/L-DOPA	20	↑	ND	ND	Santini et al., 2010
**NUCLEUS ACCUMBENS**							
	Mouse	Quinelorane	0.63	=	↑	ND	Salles et al., 2013
		Cocaine	15^*^	↑	ND	ND	Sutton and Caron, 2015
	Rat	Quinelorane	0.16	=	↑	ND	Salles et al., 2013
		Ketamine	5	↑	ND	ND	Tedesco et al., 2013
		Cocaine	15	↑ (NS)	↑ (NS)	ND	Wu et al., 2011
		NMDA	250 (ng) icv	↑ (NS)	↑ (NS)	ND	Wang et al., 2010
**CORTEX**							
*Prefrontal*	Mouse	WAY181187	10	ND	↑	ND	Meffre et al., 2012
	Rat	NMDA	500 (ng) icv	↑ (NS)	↑ (NS)	ND	Yu et al., 2013
		WAY181187	10	ND	↑	ND	Meffre et al., 2012
		MK801	2	ND	↓	ND	Yoon et al., 2008
			1^*^	ND	↑	ND	Yoon et al., 2008
			1^*^	ND	↓	Principal cells	Kim et al., 2010
		Oubain	1 (mM) icv	↑	↑	Principal cells	Kim et al., 2013
*Cingulate*		MK801	1^*^	ND	↓	Principal cells	Kim et al., 2010
		Oubain	1 (mM) icv	↑	↑	Principal cells	Kim et al., 2013
*Insular*		MK801	1^*^	ND	↓	Principal cells	Kim et al., 2010
*Prelimbic*		Ketamine	5	↑	ND	ND	Tedesco et al., 2013
			10	↑	ND	ND	Tedesco et al., 2013
*Infralimbic*			5	↑	ND	ND	Tedesco et al., 2013
			10	↑	ND	ND	Tedesco et al., 2013
*Somatosensory*		Kainate	10	↑	ND	pyramidal cells	Macias et al., 2013
*Piriform*				↑	ND	ND	Macias et al., 2013
*NS*			12	ND	↑	ND	Chen et al., 2012
				↑ (NS)	↑ (NS)	ND	Zeng et al., 2009
		Pilocarpine	300	↑ (NS)	↑ (NS)	ND	Huang et al., 2010
		PTZ	75	↑	↑	ND	Zhang and Wong, 2012
		Cocaine	15	↑ (NS)	↑ (NS)	ND	Wu et al., 2011
		MK801	0.5	↓	↓	ND	Kim et al., 2010
			1	↓	↓	ND	Kim et al., 2010
		L-phenylalanine	2 (mg/g)	↓ (NS)	↓ (NS)	ND	Roberts and Morelos, 1979
**VENTRAL TEGMENTAL AREA**							
	Mouse	Morphine	25 (pellet)^*^	↑ (NS)	↑ (NS)	TH+ cells	Mazei-Robison et al., 2011
	Rat	Cocaine	15	↑ (NS)	↑ (NS)	ND	Wu et al., 2011
**AMYGDALA**							
*Basolateral*	Rat	Ketamine	10	↑	ND	ND	Tedesco et al., 2013
*NS*		Kainate	10	↑	ND	ND	Macias et al., 2013
**HYPOTHALAMUS**							
*Arcuate*	Mouse	Olanzapine	20	↑	ND	ND	Knight et al., 2012
		Ghrelin	66 (mg)	↑	↑	NPY+ cells	Knight et al., 2012
			6 (mg) icv	ND	↑	ND	Villanueva et al., 2009
		Leptin	5 (mg) icv	ND	↑	ND	Gong et al., 2007
		Insulin	300 (mU/ml) icv	ND	↑	ND	Villanueva et al., 2009
			400 (mU/ml)	ND	↑	ND	Villanueva et al., 2009
*Ventromedial*			300 (mU/ml) icv	ND	↑	ND	Villanueva et al., 2009
*Paraventricular*		Clozapine	10	↑	ND	ND	Knight et al., 2012
	Rat	Leptin	10 (mg) icv	ND	↑	ND	Cota et al., 2006, 2008
*NS*		CNTFAx15	1.5 (mg) icv	ND	↑	ND	Cota et al., 2008
		C75	30 (mg) icv	ND	↑	ND	Proulx et al., 2008
		Cerulenin	90 (mg) icv	ND	↑	ND	Proulx et al., 2008

When not specified, a single dose of drug was administered;

* *repeated doses of drug were administered. NS, not specified; ND, not determined; DG, Dentate Gyrus; MSNs, Medium-sized Spiny Neurons; THC, tetrahydrocannabinol; PTZ, Pentylenetetrazol; 6-OHDA, 6-hydroxydopamine; L-DOPA, levodopa; MPTP, 1-methyl-4-phenyl-1,2,3,6-tetrahydropyridine*.

### Physiological and pathophysiological conditions

Since the pioneering report demonstrating that rpS6 phosphorylation was enhanced in the CA1 subfield of the hippocampus in mice trained to contextual fear conditioning (Kelleher et al., 2004), rpS6 phosphorylation has been used as a marker of neuronal and circuit activation following physiological conditions (Table 3). Thus, phospho-rpS6 levels oscillate in the hippocampus and the suprachiasmatic nucleus of the hypothalamus along the circadian cycle (Table 3). rpS6 phosphorylation is also strongly modulated in the amygdala and the hypothalamus when defensive behaviors (freezing, escape or attacks) are engaged or in the hypothalamus in response to nutritional perturbations (Table 3). Finally, altered rpS6 phosphorylation has been reported in rodents following spontaneous seizures and traumatic brain injury and in humans in several neurodevelopmental disorders including Down syndrome, Tuberous sclerosis, Autism, and Rett syndrome (Table 3).

**Table 3:** **rpS6 phosphorylation regulation under physiological and pathophysiological conditions**.

**Brain areas**	**Species**	**Model**	**S235/236**	**S240/244**	**References**
**HIPPOCAMPUS**					
	Mouse	Contextual FC	↑	ND	Kelleher et al., 2004; Saraf et al., 2014
		Circadian cycle	↑	ND	Saraf et al., 2014
	Rat	Pilocarpine-induced spontaneous seizure	↑ (NS)	↑ (NS)	Huang et al., 2010; Saraf et al., 2014
		Kainate-induced spontaneous seizure	↑	↑	Zeng et al., 2009
		Kainate-induced early life seizure	=	↑	Bernard et al., 2013
		Traumatic brain injury	↑	ND	Chen et al., 2007
	Human	Down syndrome	↑ (NS)	↑ (NS)	Iyer et al., 2014
		Alzheimer's disease (severe-stage)	↑	ND	Sun et al., 2014
**STRIATUM**					
	Mouse	Stroke	ND	↑	Xiong et al., 2014
**NUCLEUS ACCUMBENS**					
	Mouse	Highly palatable isocaloric food	↑	ND	Guegan et al., 2013
	Rat	Cue-induced cocaine reinstatement	↑	ND	Wang et al., 2010
**CORTEX**					
*Prefrontal*	Mouse	Highly palatable isocaloric food	↑	ND	Guegan et al., 2013
*NS*		Middle cerebral artery occlusion	ND	↑	Xiong et al., 2014
*Prefrontal*	Rat	Focal cerebral ischemia	↓	ND	Koh, 2013
		Neonatal phencyclidine	ND	↑	Meffre et al., 2012
		Social isolation	ND	↑	Meffre et al., 2012
*Prelimbic*	Rat	Cue-induced alcohol reinstatement	↑	ND	Barak et al., 2013
		Extinction after retrieval of FC	↑	ND	Tedesco et al., 2014
*Infralimbic*		Extinction after retrieval of FC	↑	ND	Tedesco et al., 2014
*Orbitofrontal*		Cue-induced alcohol reinstatement	↑	ND	Barak et al., 2013
*Parietal*		Traumatic brain injury	↑	ND	Chen et al., 2007
*NS*		Pilocarpine-induced spontaneous seizure	↑ (NS)	↑ (NS)	Huang et al., 2010
		Kainate-induced spontaneous seizure	↑ (NS)	↑ (NS)	Zeng et al., 2009
*Cortex*	Human	Focal cortical dysplasia	↑	=	Baybis et al., 2004
		Focal cortical dysplasia	↑	ND	Jansen et al., 2015
		Hemimegalencephaly	↑	ND	Jansen et al., 2015
*Cortical Tubers*		Tuberous sclerosis (unspecified)	↑	=	Baybis et al., 2004
		Tuberous sclerosis (specific mutations)	↑	ND	Parker et al., 2011
		Tuberous sclerosis (TSC1 mutation)	↑ (NS)	↑ (NS)	Jansen et al., 2004
*Frontal cortex*		Hemimegalencephaly	↑	ND	Aronica et al., 2007
*Medial Temporal Cortex*		Alzheimer's disease	↑	=	An et al., 2003
**AMYGDALA**					
*Central*	Rat	Cue-induced alcohol reinstatement	↑	ND	Barak et al., 2013
*Basolateral*		Extinction after retrieval of FC	↑	ND	Tedesco et al., 2014
**HYPOTHALAMUS**					
*Paraventricular*	Mouse	Salt	↑	↑	Knight et al., 2012
		Leucine deprivation	↓	ND	Xia et al., 2012
*Arcuate*		Fasting	ND	↑	Knight et al., 2012
		Fasted	ND	↑	Villanueva et al., 2009
		Leucine deprivation	↓	ND	Xia et al., 2012
*Dorsomedial*			ND	↑	Knight et al., 2012
*Preoptic area*			ND	↑	Knight et al., 2012
*Ventrolateral*		Resident-intruder	↑	↑	Knight et al., 2012
*Premammillary*		Cat odor	ND	↑	Knight et al., 2012
*Suprachiasmatic*		Light	↑	↑	Cao et al., 2011; Knight et al., 2012
		Circadian cycle	ND	↑	Cao et al., 2011
*Supraoptic*		Dehydration	ND	↑	Knight et al., 2012
		Salt	ND	↑	Knight et al., 2012
*Arcuate*	Rat	Fasted	ND	↓	Cota et al., 2006
**PERIAQUEDUCTAL GRAY**					
	Mouse	Resident-intruder	↑	ND	Knight et al., 2012
**SPINAL CORD**					
	Rat	Inflammatory pain (carrageenan)	↑	ND	Norsted Gregory et al., 2010
		Neurogenic inflammation (capsaicin)	↑	ND	Géranton et al., 2009
**DORSAL ROOT GANGLIA**					
	Rat	Inflammatory pain (carrageenan)	↑	ND	Norsted Gregory et al., 2010
		Neuropathic pain (SNI)	↑	ND	Géranton et al., 2009
NS	Human	Glioblastoma	↑	=	Harter et al., 2015
		SEGA	↑	ND	Chan et al., 2004

*NS, not specified; ND, not determined; FC, fear conditioning; SEGA, subependymal giant cell astrocytoma*.

### Genetic mouse models displaying altered rpS6 phosphorylation

Most of the full or conditional knockout mice for the key components of the mTORC1 pathway display altered rpS6 phosphorylation (Table 4). Interestingly, the vast majority of mutant mice in which dysregulation of rpS6 phosphorylation has been demonstrated correspond to mouse models for various neurological and neurodevelopmental disorders, including Tuberous sclerosis, Down syndrome, Rett syndrome, Angelman syndrome, and Fragile X syndrome, among others. Most of these pathologies share common features such as autism, intellectual disability, and epilepsy, which might be rescued by mTORC1 inhibitors under certain circumstances. The phosphorylation of rpS6 is also altered in neurodegenerative diseases such as Huntington disease and in mouse models of psychiatric disorders such as schizophrenia (Table 4). Other genetic mouse models showing hormonal perturbations as leptin deficiency also display altered rpS6 phosphorylation in the hypothalamus (Table 4).

**Table 4:** **rpS6 phosphorylation in genetic mouse models**.

**Model**	**Brain area/cell-type**	**S235/236**	**S240/244**	**References**
**KNOCKOUT**				
Akt3	Whole brain	↓	↓	Easton et al., 2005
Tsc2^+∕−^	Hippocampus	↑	ND	Ehninger et al., 2008
Hdc	Striatum	↑	=	Rapanelli et al., 2014
Fmr1	Hippocampus/pyramidal cells	↑	↑	Bhattacharya et al., 2012
S6K2	Hippocampus	↓	=	Antion et al., 2008a
Mecp2	Cortex/principal cells	↓	↓	Ricciardi et al., 2011
	Cerebellum	↓	↓	Ricciardi et al., 2011
	Hippocampus	↓	=	Ricciardi et al., 2011
miR-199a-2	Cortex/principal cells	↓	ND	Tsujimura et al., 2015
	Hippocampus/pyramidal cells	↓	ND	Tsujimura et al., 2015
Cdkl5	Cortex/principal cells	↓	↓	Amendola et al., 2014
DAT	Nucleus accumbens	↑	ND	Sutton and Caron, 2015
Lep^ob∕ob^	Hypothalamus (arcuate)	ND	↑	Villanueva et al., 2009
Lepr^db∕db^	Hypothalamus (arcuate)	ND	↑	Villanueva et al., 2009
**CONDITIONAL KNOCKOUT**				
Tsc1^Syn1^	Cortex/principal cells	ND	↑	Meikle et al., 2007
	Hippocampus	ND	↑	Meikle et al., 2007
Tsc1^GFAP^	Cortex	↑	ND	Parker et al., 2011
	Cortex	↑ (NS)	↑ (NS)	Zeng et al., 2008, 2011
	Hippocampus	↑ (NS)	↑ (NS)	Zeng et al., 2008, 2011
Tsc1^AAV−CreGFP^	Hippocampus/pyramidal cells	ND	↑	Bateup et al., 2011
Tsc1^MCH^	Hypothalamus	↑	ND	Knight et al., 2012
Tsc1^L7^	Cerebellum/purkinje cells	↑ (NS)	↑ (NS)	Tsai et al., 2012
Tsc1^Emx1^	Cortex/principal cells	↑	ND	Magri et al., 2011
Tsc1^CaMKII^	Cortex	↑	ND	McMahon et al., 2012
	Hippocampus	↑	ND	McMahon et al., 2012
Tsc1^Temporal^^#^	Cortex	ND	↑	Feliciano et al., 2011
Tsc2^GFAP1^	Cortex	↑ (NS)	↑ (NS)	Zeng et al., 2011
	Hippocampus	↑ (NS)	↑ (NS)	Zeng et al., 2011
Tsc2^hGFAP^	Cortex/principal cells	ND	↑	Way et al., 2009
	Hippocampus/principal cells	ND	↑	Way et al., 2009
Tsc2^CaMKII^	Hippocampus/principal cells	ND	↑	Rozas et al., 2015
Rheb1^Nestin^	Hypothalamus	↓	↓	Zou et al., 2011; Yang et al., 2014
	Cortex	↓	↓	Zou et al., 2011
	Cerebellum	↓	↓	Zou et al., 2011
	Hippocampus	↓	↓	Zou et al., 2011
Rheb^GFAP^	Hippocampus/granule cells	ND	↑	Banerjee et al., 2011
Pten^GFAP^^*^	Hippocampus/granule cells	↑	↑	Kwon et al., 2003; Chalhoub et al., 2006; Lugo et al., 2013
	Cerebellum	↑	ND	Kwon et al., 2003
Pten^GFAP^^*^/S6K1 ko	Hippocampus/granule cells	↑	ND	Chalhoub et al., 2006
Pten^Nestin^	Hippocampus/granule cells	↑	ND	Kwon et al., 2006; Zhou et al., 2009
	Cortex	↑	ND	Zhou et al., 2009
Pten^DATCreERT2^	Ventral midbrain/DA neurons	↑	ND	Domanskyi et al., 2011
Rictor^Nestin^	Whole brain	=	ND	Thomanetz et al., 2013
Dicer1^CaMKCreERT2^	Hypothalamus/arcuate nucleus	↑	ND	Vinnikov et al., 2014
**TARGETED MUTATION**				
Ube3^Atm1Alb∕J^	Cerebellum/purkinje cells	↑	↑	Sun et al., 2015
Tsc2DRG	Hippocampus	↑	=	Chévere-Torres et al., 2012
Ts1Cje	Hippocampus/principal cells	↑	ND	Troca-Marín et al., 2011
N171-N82Q	Striatum	↓	ND	Ravikumar et al., 2004; Lee et al., 2015
	Cortex	↓	ND	Ravikumar et al., 2004
	Cortex	↓	=	Fox et al., 2010
3xTg-AD	Whole brain	↑	ND	Caccamo et al., 2015
**VIRAL-MEDIATED GENE TRANSFER**				
DISC1-shRNA	Hippocampus/granule cells	↑	ND	Kim et al., 2009; Zhou et al., 2013
S6K1^CA^	Prefrontal cortex	↑	ND	Dwyer et al., 2015
S6K1^DN^	Prefrontal cortex	↑	ND	Dwyer et al., 2015

* *The Gfap-Cre mice used in these studies lead to Pten deletion in a subset of neuronal cells, including the majority of granule cells in the dentate gyrus and cerebellum*.

# *In utero electroporation to express Cre recombinase and remove Tsc1 in Tsc1^floxed∕mutant^ heterozygous embryos at E16. NS, not specified; ND, not determined; DA, dopaminergic*.

## Physiological role of rpS6 phosphorylation

### Role in overall mRNA translation

Despite the growing number of reports analyzing the phosphorylation of rpS6, its biological significance still remains controversial. One of the first hypotheses put forward suggested that rpS6 phosphorylation played a role in translation initiation. Thus, an early *in vitro* study reported a correlation between the phosphorylation of rpS6 and enhanced translation under certain experimental conditions (Thomas et al., 1982). Moreover, the 40S subunit with a highest proportion of phosphorylated rpS6 was preferentially found into polysomes compared to subpolysomal fractions (Duncan and McConkey, 1982). However, several studies rapidly called into question this hypothesis. Indeed, although localized at the mRNA/tRNA binding site junction between the small and large ribosomal subunits (Nygård and Nilsson, 1990), increased rpS6 phosphorylation is not sufficient to mobilize small ribosomal subunits into protein synthesis (Kruppa and Clemens, 1984; Tas and Martini, 1987). Finally, the generation of rpS6 knockin mice, in which the five phosphorylated serines were replaced by alanines, constituted a valuable tool to determine whether rpS6 phosphorylation and the protein synthesis were causally linked (Ruvinsky et al., 2005). Unexpectedly, protein synthesis is increased in mouse embryo fibroblasts (MEF) of phospho-deficient mice. Moreover, a similar (Ruvinsky et al., 2005) or even increased (Chauvin et al., 2014) proportion of ribosomes engaged in translation were found in the liver of rpS6 knockin mice. Together, these puzzling observations suggest a negative role of rpS6 phosphorylation on global protein synthesis or the presence of feedback mechanisms taking place in this mouse model.

### Role in TOP mRNA translation

The phosphorylation of rpS6 through the mTORC1/S6K axis was believed for many years to exert an effect on the translation of a specific subset of mRNAs bearing a 5′ terminal oligopyrimidine tract (TOP). However, this long-lasting model has been challenged by subsequent studies showing that MEFs from the double mutant S6K1/2 as well as from the rpS6 knockin mouse lines exhibit normal TOP translation (Tang et al., 2001; Stolovich et al., 2002; Ruvinsky et al., 2005). Further work demonstrated that insulin-induced TOP translation requires the PI3K/TSC/Rheb/mTOR pathway but is independent of the S6K/rpS6 axis (Patursky-Polischuk et al., 2009).

Despite these evidences, the involvement of rpS6 phosphorylation in the control of translation in the nervous system is still controversial. Indeed, several findings in hippocampal neurons and slices correlated increased rpS6 phosphorylation with enhanced global and TOP-encoded protein synthesis following different forms of synaptic plasticity (Kelleher et al., 2004; Klann and Dever, 2004; Tsokas et al., 2005, 2007; Antion et al., 2008a,b) or in a mouse model of fragile X syndrome (Bhattacharya et al., 2012). By contrast, such correlations have not been observed *in vivo* in the striatum where the pharmacologically-induced rpS6 phosphorylation by d-amphetamine, haloperidol, or papaverine relies on the activation of the cAMP/PKA/DARPP-32 pathway (Biever et al., 2015). Interestingly, the direct binding of mRNA to the small ribosomal subunit decreases or increases after cAMP-dependent or cAMP-independent phosphorylation of rpS6, respectively (Burkhard and Traugh, 1983). Therefore, one cannot exclude that when an upstream signaling cascade other than PKA is engaged, for example mTORC1/S6K or ERK/RSK, rpS6 phosphorylation might have different physiological roles. Nevertheless, despite compelling studies indicating that rpS6 phosphorylation is dispensable for efficient global and TOP mRNA translation, the role of the phosphorylation in the translation of a specific subset of mRNAs remains to be fully addressed by high-throughput sequencing analyses of total and polysomal RNAs combined with proteomic approaches.

### Extratranslational functions

Another possibility is that rpS6 phosphorylation, within or outside the ribosome, exerts functions unrelated to mRNA translation, for example by interacting with other cellular proteins, which might become active or inactive upon the binding with rpS6. Indeed, co-immunoprecipitation studies suggest either a direct or indirect interaction of rpS6 with several extraribosomal proteins, including heat-shock protein 90 (Kim et al., 2006), alphavirus non-structural protein (Montgomery et al., 2006), DAPK (Schumacher et al., 2006), huntingtin (Culver et al., 2012), and mTOR complex 2 (mTORC2) (Yano et al., 2014). In the latter, rpS6 phosphorylation has been proposed to have a role in cardioprotective signaling by amplifying mTORC2-mediated Akt phosphorylation (Yano et al., 2014). In the mouse liver, Chauvin and colleagues recently uncovered the involvement of rpS6 phosphorylation in the control of the ribosome biogenesis (RiBi) transcriptional program by S6Ks (Chauvin et al., 2014). This program regulates the expression of nucleolar proteins required for ribosomal RNA synthesis, cleavage, post-transcriptional modifications, ribosome assembly, and export (Lempiäinen and Shore, 2009). Whether all these translation-unrelated responses occur in the brain merits further study.

### Extraribosomal functions

Surprisingly, extraribosomal functions have been attributed to several ribosomal proteins (Wool, 1996; Warner and McIntosh, 2009). For instance, the ribosomal protein rpL13a, when phosphorylated, is released from the 60S ribosomal subunit and acts as a silencer of targeted mRNAs (Mazumder et al., 2003). In this regard, few studies suggested an extraribosomal role of rpS6 phosphorylation (Kim et al., 2014; Son et al., 2015; Xiao et al., 2015). Recent work in the plant *Arabidopsis Thaliana* proposes a role of rpS6 in rRNA synthesis and rDNA transcription via its interaction with the histone deacetylase AtHD2B (Kim et al., 2014) and the histone chaperon AtNAP1 (Son et al., 2015), respectively, an effect that might be dependent on the phosphorylation state of rpS6. Finally, the ubiquitylation and proteasomal degradation of phosphorylated rpS6 following its subsequent interaction with PALL has been identified as a critical mechanism regulating efferocytosis in drosophila (Xiao et al., 2015). To date, such extraribosomal functions of rpS6 in the nervous system have not been described.

## Concluding remarks

Since the pioneering studies performed four decades ago, many progresses have been made regarding the identification of signaling events leading to rpS6 phosphorylation. Although rpS6 phosphorylation is still considered as a readout of mTORC1/S6K activity, caution should be taken with this assumption since other intracellular cascades largely contribute to the regulation of rpS6 phosphorylation, as exemplified with the PKA/DARPP-32/PP-1 pathway in the striatum. One should also keep in mind that the different sites of phosphorylation can be regulated independently in various brain areas or different cell-types within a same brain region. Although rpS6 phosphorylation has been and will remain a valuable hallmark of neuronal activity, understanding its biological role in the brain is undoubtedly one of the major challenges of the coming years.

## Author contributions

AB, EV, and EP wrote the manuscript.

### Conflict of interest statement

The authors declare that the research was conducted in the absence of any commercial or financial relationships that could be construed as a potential conflict of interest.
